# Intra- and interpersonal emotion regulation are altered in individuals with childhood maltreatment: cross-sectional associations and effects on daily life mood

**DOI:** 10.1186/s40479-025-00297-0

**Published:** 2025-06-17

**Authors:** Inga Niedtfeld, Sara E. Schmitz, Michelle Langenstein, Johanna Hepp

**Affiliations:** 1 Department of Psychosomatic Medicine and Psychotherapy, Central Institute of Mental Health, Medical Faculty Mannheim, Heidelberg University, PO-Box 12 21 20, Mannheim, 68072 Germany; 2 Mental mHealth Lab, Institute of Sports and Sports Science, Karlsruhe Institute of Technology, Karlsruhe, Germany

**Keywords:** Childhood adversity, Child abuse, Child neglect, Emotional regulation, Ecological momentary assessment

## Abstract

**Background:**

Childhood maltreatment (CM) is a potent predictor of lifelong emotional and psychological difficulties. We investigated how CM affects intra- and interpersonal emotion regulation (ER) processes and explored the impact of these ER difficulties on daily life mood.

**Methods:**

We explored the CM-ER association in two studies. Data and code are available at https://osf.io/cbkyj/. Study 1 tested pre-registered hypotheses (https://osf.io/2kt35) on the association between CM and self-reported ER difficulties in a web-based sample (*N* = 553). Study 2 used ecological momentary assessment data (*N* = 103) to examine how trait-level intra- and interpersonal ER difficulties predict momentary negative mood in pseudo-randomized daily assessments over seven days (3,973 observations), particularly in the context of momentary interpersonal stressors.

**Results:**

We replicated a positive association between CM severity and intrapersonal ER difficulties and revealed differential effects of abuse versus neglect. Additionally, CM was associated with lower use of and more difficulties in interpersonal ER. In exploratory analyses, we found that intrapersonal ER difficulties predicted increased negative mood during interpersonal stressors, while interpersonal ER use showed no significant effects on momentary mood.

**Conclusions:**

Our findings highlight a critical treatment target: intrapersonal ER impairments, which uniquely predict daily mood fluctuations beyond the effect of CM severity. Reduced use of interpersonal ER was also observed in individuals with more severe CM, which could be adaptive in certain environments where social support is unavailable or inconsistent. We emphasize the need to prioritize ER-focused interventions in clinical settings to address the enduring consequences of CM.

**Supplementary Information:**

The online version contains supplementary material available at 10.1186/s40479-025-00297-0.

## Introduction

Childhood maltreatment (CM) has consistently been linked to negative outcomes, including increased risk for the incidence of mental disorders in adulthood [1, 2]. CM is defined as "any act or series of acts of commission or omission by a parent or other caregiver that results in possible, threatened, or actual harm to the child” ([3], S.11). A commonly used classification of CM includes the categories sexual abuse, emotional abuse (e.g., parental rejection, devaluation, or verbal abuse), physical abuse (e.g., parental physical violence), emotional neglect (e.g., failure to meet the child's basic psychological needs for support and belonging), and physical neglect (e.g., failure to provide adequate food, clothing, or basic medical care) [4]. Previous research on CM has been largely dominated by a focus on sexual abuse, while other types of CM remain under-researched, even though they account for a significant proportion of CM [5]. The importance of studying different types of CM is particularly evident when considering the high prevalence of emotional abuse and emotional neglect. Prevalence rates for physical abuse vary across continents and range from 22 to 60%, with higher prevalence rates for men in European samples. Emotional abuse exhibits prevalence rates ranging from 6 to 61%, with mixed findings regarding gender patterns. Neglect shows prevalence rates varying from 17 to 65%, with predominantly similar or higher prevalence rates for women compared to men [6]. In addition to the individual burden for those affected, CM also creates a high financial burden for society as a whole. Estimates for Germany put the total annual direct and indirect cost of CM at between 11 and 30 billion Euros for the age group from 15 up to including 64 years [7]. Due to the multitude of negative consequences that CM has, it is particularly important to investigate the pathways through which it negatively affects mental health. Herein, we focus on two possible factors: impaired intra- and interpersonal emotion regulation (ER).

*Intrapersonal ER* refers to the processes and strategies individuals use to manage and modulate their own emotions within themselves. This involves the internal regulation of one's emotional experiences, expression, and physiological responses. According to Gratz and Roemer [8], adaptive intrapersonal ER comprises several components in terms of how emotions are processed and how behavioral consequences play out. With regard to emotion processing, components of adaptive intrapersonal ER are emotional clarity, understanding of emotions, acceptance of the current emotional state, and attention to one's own emotions. With regard to behavioral consequences, goal-directed behavior despite prevailing negative emotions, impulse control, and the flexible use of adaptive ER strategies are described.

Research in samples with CM history suggests a robust link between CM and deficits in intrapersonal ER (see meta-analysis by [9]). More specifically, several components of adaptive intrapersonal ER as described by Gratz and Roemer appear to be impaired in these individuals and this appears to depend on the type of CM that was experienced. *Childhood neglect* was shown to be associated with reduced emotional clarity [10] and understanding of own emotions [11] – both components of adaptive emotion processing [8]. In addition, studies also showed an association between a history of childhood neglect and lack of adaptive ER strategy use [12–14], which is a central behavioral component of adaptive ER. This led McLaughlin, Sheridan [15] to suggest that, in the case of neglect, the lack of complex, age-appropriate cognitive and social input likely impairs development of these central intrapersonal ER components. In the case of *childhood abuse*, the experience of threat to one’s physical integrity is critical [15]. In line with this, increased emotional reactivity and impulse control problems have been observed in abused children [16, 17].

Taken together, previous findings suggest that CM is associated with difficulties in intrapersonal ER and, in terms of the model by Gratz and Roemer, these unfold both at an emotion processing and a behavioral level. Importantly, such intrapersonal ER difficulties were shown to moderate the association between CM and mental health problems in adulthood, as summarized in a recent meta-analysis [9]. Thus, impaired intrapersonal ER following CM may be a pathway to later psychopathology and at the same time a potential treatment target that warrants further investigation. The first aim of the present study was therefore to investigate the association between CM and intrapersonal ER difficulties. Specifically, we aimed to assess the associations between different types of CM (neglect and abuse) with components of intrapersonal ER as described by Gratz and Roemer.

While there is substantial evidence on intrapersonal emotion regulation and CM, very little research has been conducted on *inter*personal ER [18]. More specifically, *intrinsic interpersonal ER* is the process of turning to other people for help and support in modulating one's own emotions. Interpersonal ER is traditionally investigated in developmental psychology due to its high importance in childhood, as ER largely occurs through the actions and intervention of caregivers during early years [19, 20]. In the context of CM, interpersonal ER is likely impaired – but direct evidence is lacking. Previous work suggests that caregivers of traumatized children tend to interact with them in an insensitive, unpredictable or even threatening way [21], which may result in altered emotion socialization (i.e., the process by which children learn to understand and regulate emotions through interactions with caregivers) [17, 21, 22]. Consequently, adaptive interpersonal ER strategies likely cannot be acquired. Experimental work supports this, showing that negative parental reactions (i.e. punitive, minimizing, distressed, or upset) during stressful situations are associated with decreased levels of venting in children [23]. Translating this finding to the context of CM, one could assume that exposed children might show difficulties in expressing negative emotions to others as a result of repeated inadequate responses by their caregivers to own feelings of distress and helplessness.

As with intrapersonal ER, it appears likely that difficulties in interpersonal ER could mediate the association between CM and psychopathology later in life and thus present a treatment target. Evidence on PTSD development supports this notion, as a lack of social support (which can be seen as a variant of interpersonal emotion regulation; [18]) after traumatic experiences is a strong risk factor for PTSD development [24, 25]. Consequently, the second aim of the study was to investigate the association between CM and interpersonal ER.

In addition to probing whether CM is associated with impaired intra- and interpersonal ER at a between-person level (i.e. whether individuals with higher levels of CM also show greater impairment in ER, aims 1 and 2), the third aim of the present study was to assess whether ER difficulties in individuals with CM have daily life correlates. Specifically, we aimed to investigate whether (trait-level) intra- and interpersonal ER difficulties predict how negatively individuals with CM feel when experiencing an interpersonal stressor in daily life. Previous studies showed that flexible use of adaptive ER strategies was related to less daily life negative affect [26, 27] and greater well-being [28]. As both intra- and interpersonal ER strategy use tend to be impaired in individuals with CM, negative affect in daily life should increase with higher levels of CM. Interpersonal stressors are an important context factor for this, as they are a potent triggers for negative affect in daily life (e.g., [29–32]) and also inherently relevant to investigating *inter*personal ER. Thus, in addition to adopting a between-person perspective for aims 1 and 2, aim 3 comprised a within-person element in that we aimed to investigate the effects of ER difficulties on the interpersonal stressor-negative affect link in daily life.

### The current studies

We investigated the three research aims using two methodological approaches: cross-sectional self-reports and ambulatory assessment (AA, [33]) to collect daily-life data. In study 1, we tested pre-registered hypotheses (https://osf.io/2kt35) on the association between different types of CM and intrapersonal as well as interpersonal ER. In study 2, we re-analyzed an existing AA dataset [34, 35] to examine the effects of ER difficulties on the link between interpersonal stressors and momentary mood in daily life.

Based on the above reviewed evidence, we derived a number of a priori hypotheses. Grounded in theoretical consideration that families where CM occurs provide an inadequate context for the development of adaptive ER strategies [19, 20], and based on previous studies demonstrating this association empirically [17, 21, 22, 36], we hypothesized that CM is positively associated with self-reported difficulties in intrapersonal ER (H1).

In addition to hypothesizing a global association between CM and intrapersonal ER, we derived hypotheses for specific types of CM and intrapersonal ER components based on the model by Gratz and Roemer. Specifically, we focused on hypotheses contrasting neglect versus abuse, aiming to replicate previous empirical findings on differential effects of these two types of CM. Following findings on increased emotional reactivity and impulse control problems in abused children [16, 17], we hypothesized that higher levels of childhood abuse (i.e. emotional abuse or physical abuse) are associated with greater difficulties with impulsivity in intrapersonal ER, beyond any effects of childhood neglect (H2.1). For Hypothesis H2.1, we decided to exclude participants who reported sexual abuse from the respective analysis, to avoid confounding results with the deleterious and well-researched effects of sexual abuse. For childhood neglect (i.e. emotional neglect or physical neglect), we hypothesized associations with a limited access to intrapersonal ER strategies (H2.2 A, following [12–14]), lower emotional awareness (H2.2B, following [10]) and lower emotional clarity (H2.2 C, following [11]), each above and beyond any effects of childhood abuse.

For interpersonal ER, we chose to formulate non-directional hypotheses due to lack of previous work on associations with CM. We hypothesized that the level of interpersonal ER use varies depending on the level of CM (H3.1) and that difficulties in interpersonal ER also vary depending on the level of CM (H3.2).

With regard to the daily life context, we hypothesized that (H4.1) difficulties in intrapersonal ER positively predict daily life negative mood beyond any effects of CM, and that (H4.2) this association is stronger in the context of interpersonal stressors. Additionally, we hypothesized that (H5.1) interpersonal ER use predicts daily life negative mood beyond any effects of CM, and that (H5.2) this association is stronger in the context of interpersonal stressors.

## Methods study 1

Study 1 was pre-registered at https://osf.io/2kt35, and the preregistration protocol was published online before data collection began on February 8, 2021. All data and code behind this analysis has been made publicly available and can be accessed at https://osf.io/cbkyj/. Ethics approval for the study was granted by the Ethics Committee of the Medical Faculty Mannheim at Heidelberg University (protocol no. 2018–588 N-MA). We conducted a web-based survey using the platform www.soscisurvey.de that we advertised on social media. To advertise the study on Instagram, we contacted individuals who posted regularly about mental health topics, and who had a significant reach (> 3500 followers) and asked them to make the study link available to all subscribers for 24 hours. In addition, the link to the online survey was shared in various online forums dedicated to mental health, and on our department’s website. At the beginning of the study, participants had to provide informed consent by selecting a checkbox indicating that they understood the contents of the study and data protection regulations, and that they were at least 18 years of age. In total, *n *= 492 participants completed the entire survey. As pre-registered, we pooled data from the web-based sample with self-report data from *n* = 61 individuals recruited within a research training group (www.grk2350.de) on psychosocial consequences of traumatic childhood experiences (see [35]), to ensure that individuals with high levels of CM would be represented.

### Childhood maltreatment

We assessed CM with the Childhood Trauma Questionnaire (CTQ, [4]), a retrospective self-report questionnaire measuring five types of trauma experienced before the age of 18 (emotional abuse, physical abuse, sexual abuse, emotional neglect and physical neglect) with five items each, and an openness scale with three items. The CTQ is one of the most widely used instruments for assessing the frequency of exposure to CM and shows good test–retest reliability, internal consistency, convergent and discriminant validity [37, 38]. Participants indicate frequency for each item on a Likert-type scale ranging from 1 (not at all) to 5 (very often). To determine the general frequency of exposure to childhood trauma, we computed a sum score of the five CM subscales, with higher values indicating higher trauma severity. To examine the differential effects of abuse vs. neglect, we computed a sum score for physical abuse and emotional abuse subscales (10 items). Additionally, we computed a sum score for childhood neglect based on the physical neglect and emotional neglect subscales (10 items). Note that, when testing the differential effects of (emotional and physical) abuse versus (emotional and physical) neglect (Hypothesis 2), we excluded participants who reported sexual abuse to avoid confounding results with the deleterious and well-researched effects of sexual abuse.

The internal consistency of the CTQ total score was excellent (Cronbach’s α = 0.94), as was that for the relevant subscales: abuse (α = 0.90) and neglect (α = 0.90), supporting the reliability of the scale in the present study.

### Difficulties in intrapersonal ER

Difficulties in intrapersonal emotion regulation were assessed with the well-validated Difficulties in Emotion Regulation Scale (DERS, [8], German version by [39]). The DERS is a self-report instrument consisting of 36 items to measure the flexible use of situation-appropriate emotion regulation strategies, rated on a Likert-type scale from 1 (almost never) to 5 (almost always). The German version comprises six subscales: *lack of emotional awareness, lack of emotional clarity, non-acceptance of emotional reactions, problems with goal-directed behavior, impulse control problems,* and *limited access to functional emotion regulation strategies*. In accordance with recommendations by Gratz and Roemer, we calculated sum scores for each subscale and for the overall scale, with higher values indicating more emotion regulation difficulties. The DERS total scale demonstrated excellent internal consistency in the present study (Cronbach’s α = 0.96). The internal consistency for the subscales was excellent for impulse control problems (α = 0.91), limited access to effective emotion regulation strategies (α = 0.90), and non-acceptance of emotional reactions (α = 0.91). It was very good for the subscales lack of emotional awareness (α = 0.85), lack of emotional clarity (α = 0.89), and problems with goal-directed behavior (α = 0.88).

### Interpersonal ER

To assess interpersonal ER use, we used the Interpersonal Emotion Regulation Questionnaire (IERQ [40], German version by [41]), which measures intrinsic interpersonal emotion regulation, that is, the extent to which one involves others in one's own regulation processes. The IERQ comprises 20 items reflecting four subscales *(enhancing positive affect, perspective taking, soothing, social modeling*). For the present study, we computed a sum score from all 20 items as recommended by [40], with higher values indicating greater use of interpersonal ER. The IERQ total scale demonstrated very good internal consistency in the present study (Cronbach’s α = 0.89).

### Difficulties in interpersonal ER

We included a German version of the Difficulties in Interpersonal Regulation of Emotions (DIRE) questionnaire [42]. The DIRE was translated in-house. Translation from English to German was conducted by two native German speakers with clinical expertise (ML and IN), and an independent native English-speaking translator translated this German version back into English. The DIRE is a scenario-based measure, capturing two *inter*personal ER strategies (*excessive reassurance-seeking, excessive venting*) and two *intra*personal ER strategies (*avoidance and distraction, acceptance and awareness*). Herein, we focused on the subscales excessive reassurance-seeking and excessive venting, because we already had a comprehensive and well-validated measure of intrapersonal ER difficulties (DERS). To avoid redundancy and since the German version of the DERS is validated and widely used, we chose not to include the intrapersonal subscales of the DIRE.

Three scenarios of a problematic situation are described in the DIRE: feeling upset about a time-sensitive project that needs to be completed for school/work, fighting with significant other, thinking that friends have been avoiding you. In response to each scenario, participants rate how likely they would be to use one of the following ER strategies. In line with recommendations by Dixon-Gordon, Haliczer [42], we calculated sum scores for the DIRE subscales venting (Cronbach’s α = 0.68) and reassurance seeking (Cronbach’s α = 0.83), as they refer to interpersonal ER.

### Data analysis study 1

All analyses were conducted using R, and open data and analysis code are available at https://osf.io/cbkyj. We first examined descriptive statistics and bivariate correlations for all primary variables, which are reported in Supplemental Table 1. To test the study hypotheses, we conducted a series of multiple linear regression analyses with childhood maltreatment as the primary predictor and relevant covariates (age, gender, and education) included in all models. Gender was coded as male = 1, female = 0, and education was coded as 1 for university entrance qualification (German'Abitur', completed after 12–13 years of schooling and allows entry into university studies), 0 otherwise. The CTQ sum score was used in hypotheses 1 and 3 as the primary measure of CM. In analyses pertaining to hypothesis 2, distinguishing between childhood abuse and neglect while controlling for their shared variance, we used indexes for CTQ abuse (i.e. sum score of CTQ emotional abuse and CTQ physical abuse subscales) and CTQ neglect (i.e. sum score of CTQ emotional neglect and physical neglect subscales).

To test Hypothesis 1, we examined whether higher levels of CM (CTQ sum score) were associated with more self-reported difficulties in intrapersonal emotion regulation (ER) (DERS sum score). We conducted a linear regression model predicting DERS total scores from CTQ sum scores and covariates (age, gender, education).

For Hypothesis 2, we examined differential associations of childhood abuse and neglect with intrapersonal ER difficulties, while controlling for the other maltreatment type. To isolate the effects of abuse and neglect, we excluded 259 participants (46.8% of the total sample) who had reported any experience of sexual abuse (i.e., CTQ sexual abuse subscale score > 5), leaving 294 participants for these specific analyses, ensuring that results were not confounded by the well-documented effects of sexual abuse. To test hypothesis H2.1, we conducted a linear regression model predicting impulse control difficulties (DERS impulse control subscale) from CTQ abuse and neglect scores while controlling for age, gender, and education. To test hypothesis H2.2, we conducted three separate regression models to test whether childhood abuse or neglect differentially predicted specific difficulties in intrapersonal ER, entering limited access to effective ER strategies (H2.2 A), lack of emotional awareness (H2.2B), and lack of emotional clarity (H2.2 C) as the criterion. Each model followed the same regression structure, predicting the respective DERS subscale as the dependent variable. Due to multiple comparisons, we applied a Bonferroni correction (α = 0.017\alpha = 0.017) to control for Type I error inflation.

To test Hypothesis 3, we examined the relationship between CM and interpersonal ER strategies, using multiple regression models. For hypothesis H3.1, a linear regression model was conducted predicting IERQ total score from CTQ sum score and covariates. To test hypothesis H3.2, two separate regression models examined whether higher levels of CM were associated with difficulties in interpersonal ER, specifically predicting reassurance-seeking (DIRE reassurance-seeking subscale) and venting (DIRE venting subscale). Both models followed the same structure, predicting each outcome separately.

To complement our confirmatory findings, we conducted exploratory analyses to examine the differential effects of childhood abuse vs. neglect on interpersonal ER. We replicated the structure of our confirmatory analyses (H3.1 and H3.2), but replaced the CTQ sum score with separate CTQ abuse and CTQ neglect scores, while again excluding participants with sexual abuse history. Three regression models were conducted, predicting IERQ sum score (interpersonal ER use), DIRE reassurance-seeking, and DIRE venting. All predictor variables were mean-centered prior to analysis. Model assumptions (linearity, normality of residuals, absence of multicollinearity) were checked and met for all models. Regression results are reported with standardized beta coefficients, standard errors, and confidence intervals.

## Results study 1

### Sample characteristics study 1

**Fig. 1:**
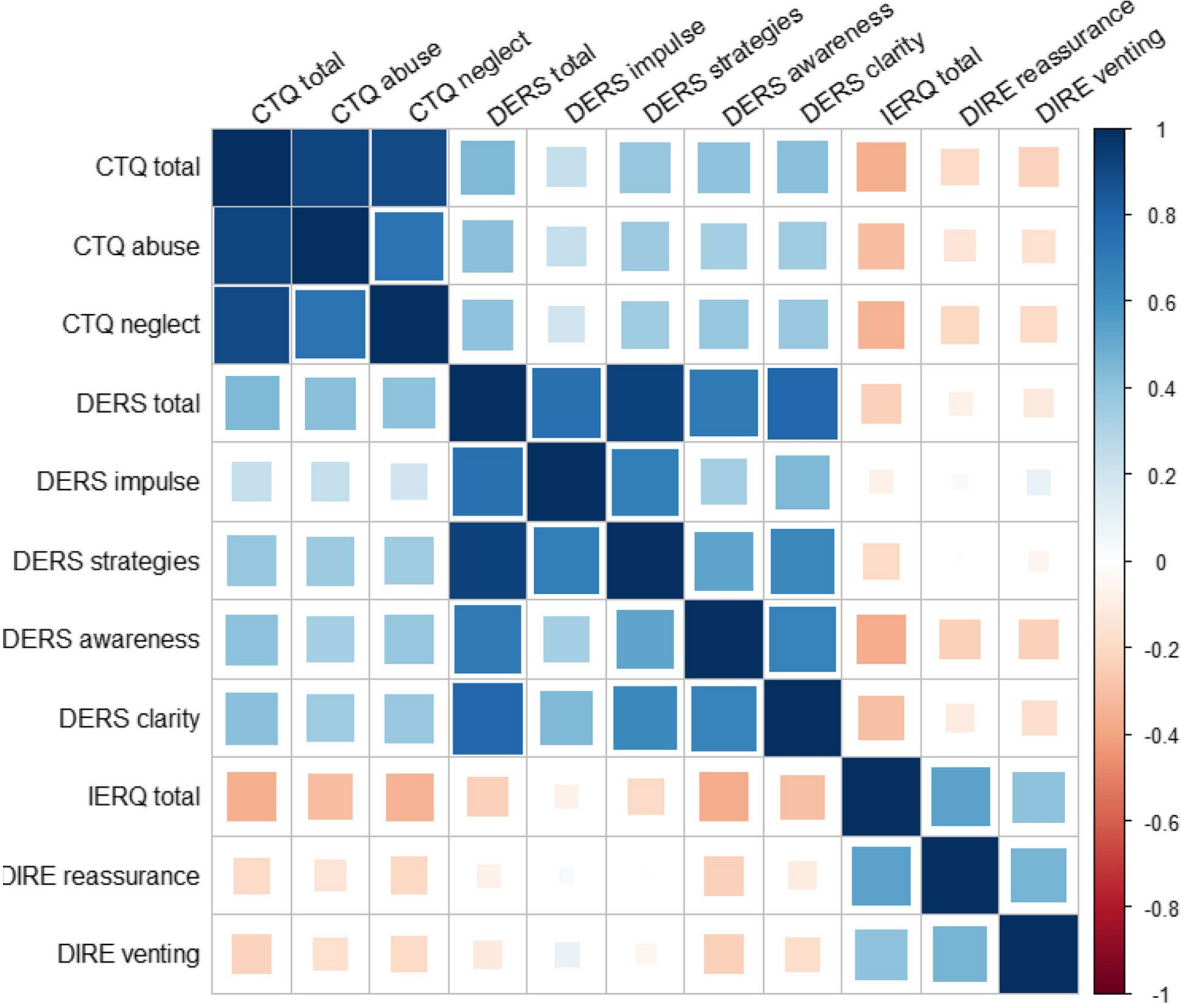
Correlation plot for central constructs in study 1, including the Childhood Trauma Questionnaire (CTQ), the Difficulties in Emotion Regulation Scale (DERS), the Interpersonal Emotion Regulation Questionnaire (IERQ), and the Difficulties in Interpersonal Regulation of Emotions (DIRE)

For data analysis, we pooled data from the web-based sample (*n*=492) with self-report data from* n *= 61 individuals recruited within a research training group (www.grk2350.de) focusing on the psychosocial consequences of traumatic childhood experiences [35]. This ensured adequate representation of individuals with high levels of childhood maltreatment (CM). The final pooled sample comprised* N* = 553 participants, ranging in age from 18 to 73 years (M = 30.11, SD = 9.39). Gender representation was predominantly cis-female (92.95%,* n* = 514), with 6.87% identifying as cis-male (*n* = 38), and one participant identifying as non-binary. In terms of formal education, 71.79% (*n* = 397) of participants held a university entrance qualification (German'Abitur'or equivalent secondary school), which is completed after 12–13 years of schooling and allows entry into university studies. More detailed descriptive results for the central constructs (CTQ, DERS, IERQ, DIRE, and their subscales) are presented in Supplemental Table 1. A correlation plot illustrating bivariate associations between CTQ (abuse and neglect scores), DERS (including relevant subscales specified in hypotheses), IERQ total score, and DIRE subscales (excessive venting and reassurance-seeking) is presented in Fig. 1.

### H1: CM as a predictor of intrapersonal ER

A multiple regression analysis tested whether higher levels of childhood maltreatment (CTQ sum score) were associated with more self-reported difficulties in intrapersonal emotion regulation (DERS sum score). Results showed a significant positive association between CTQ sum score and DERS sum score, indicating that a higher level of CM was associated with greater difficulties in intrapersonal ER.^1^Detailed results are presented in Supplemental Table 2.

### H2: Childhood neglect versus abuse as predictors of intrapersonal ER

As pre-registered, we excluded participants who reported sexual abuse from this analyses, resulting in a final sample of *n* = 294 for testing hypothesis 2.1. A multiple regression model was conducted predicting impulse control difficulties (DERS impulse control subscale) using CTQ abuse and neglect scores as predictors, while controlling for age, education, and gender. Results indicated that childhood abuse was significantly associated with greater impulse control difficulties, whereas neglect did not show a significant relationship (Table 1).

**Table 1:** Linear multiple regression model for the test of H2.1

	**DERS impulse control difficulties**				
*Predictors*	*Est*	*beta*	*SE*	*CI*	*p*
Intercept	8.27	−0.00	1.99	4.36;12.18	** < 0.001**
CTQ abuse	0.14	0.18	0.06	0.01;0.26	**0.031**
CTQ neglect	0.09	0.13	0.06	−0.02;0.21	0.116
Education	0.15	0.01	0.96	−1.75;2.05	0.877
Gender	−2.35	−0.11	1.26	−4.84;0.14	0.064
Age	0.03	0.05	0.04	−0.04;0.10	0.426

Number of observations = 264. *R*^2^ =.092. *R*^2^ adjusted =.074. Significant results *p* < .05 in bold

To test hypotheses 2.2 A, 2.2B, and 2.2 C, we conducted a series of three multiple regression models predicting limited access to effective ER strategies, lack of emotional awareness, and lack of emotional clarity. Childhood abuse was significantly associated with limited access to effective ER strategies, while childhood neglect was not. In contrast, childhood neglect was significantly associated with both lack of emotional awareness and lack of emotional clarity, whereas childhood abuse was not significantly related. Due to multiple comparisons, a Bonferroni correction (α = 0.017) was applied. Results are presented in Table 2, and an illustration of these findings is provided in Supplemental Figure 1.

**Table 2:** Linear multiple regression models for the test of H2.2

	**DERS limited access to ER strategies**				**DERS lack of emotional awareness**				**DERS lack of emotional clarity**			
*Predictors*	*Est*	*beta*	*SE*	*p*	*Est*	*beta*	*SE*	*p*	*Est*	*beta*	*SE*	*p*
Intercept	**16.81**	**0.00**	**2.68**	< .**001**	**12.85**	**0.00**	**1.91**	< **.001**	**9.46**	**0.00**	**1.53**	< .**001**
CTQ abuse	**0.26**	**0.24**	**0.08**	**.002**	0.09	0.12	0.06	.135	0.11	0.18	0.05	.019
CTQ neglect	0.17	0.16	0.08	.035	**0.14**	**0.20**	**0.06**	**.013**	**0.14**	**0.22**	**0.05**	**.004**
Education	−0.39	−0.02	1.30	.766	−0.77	−0.05	0.93	.409	−0.11	−0.01	0.74	.884
Gender	−2.32	−0.08	1.70	.175	−1.30	−0.06	1.21	.285	−1.43	−0.08	0.97	.143
Age	−0.09	−0.11	0.05	.071	−0.02	−0.03	0.04	.594	−0.06	−0.13	0.03	.027

*P*-value adjusted to.017. Individual who experienced sexual abuse were excluded from these analyses. Number of observations = 264 in each model. *R*^2^ =.163, *R*^2^ adjusted =.146 when predicting DERS limited access to ER strategies; *R*^2^ =.102, *R*^2^ adjusted =.084 when predicting DERS lack of emotional awareness; *R*^2^ =.173, *R*^2^ adjusted =.156 when predicting DERS lack of emotional clarity. Significant results *p* < .05 in bold

### H3: CM as a predictor of interpersonal ER

To test hypothesis 3.1, a multiple regression model examined whether higher levels of CM were associated with interpersonal ER use (IERQ sum score). The CTQ sum score showed a significant negative association with IERQ, indicating that individuals with higher levels of CM reported using interpersonal ER strategies less frequently (Supplemental Table 3). For hypothesis 3.2, two additional regression models tested whether CM predicted the interpersonal ER strategies of reassurance-seeking and venting [42]. Results indicated significant negative associations between CM and both strategies, suggesting that individuals with higher levels of CM were less likely to seek reassurance or vent to friends in response to stress (Supplemental Table 3, Supplemental Figure 2). Finally, hypothesis 3.3 explored differential effects of childhood abuse and neglect on interpersonal ER. These analyses indicated that neglect was significantly associated with lower interpersonal ER use and less reassurance-seeking, whereas abuse was not significantly related to any of the interpersonal ER outcomes (Table 3).

**Table 3:** Exploratory linear multiple regression models testing the association between abuse vs. neglect and interpersonal ER

	**IERQ sum score**				**DIRE excessive reassurance seeking**				**DIRE excessive venting**			
*Predictors*	*Est*	*beta*	*SE*	*p*	*Est*	*beta*	*SE*	*p*	*Est*	*beta*	*SE*	*p*
Intercept	**70.53**	**0.00**	**4.46**	< **0.001**	**17.78**	**0.00**	**2.00**	< **0.001**	**16.04**	**0.00**	**1.80**	< **0.001**
CTQ abuse	−0.24	−0.14	0.14	0.083	0.00	0.00	0.06	0.987	−0.10	−0.14	0.06	0.090
CTQ neglect	**−0.31**	**−0.18**	**0.13**	**0.023**	**−0.17**	**−0.24**	**0.06**	**0.004**	−0.02	−0.03	0.05	0.719
Education	0.29	0.01	2.17	0.893	0.44	0.03	0.97	0.654	−0.07	−0.01	0.88	0.936
Gender	−2.25	−0.05	2.84	0.429	−0.27	−0.01	1.26	0.832	−1.20	−0.07	1.14	0.291
Age	0.04	0.03	0.08	0.666	−0.03	−0.05	0.04	0.393	0.06	0.11	0.03	0.090

Individual who experienced sexual abuse were excluded from these analyses. Number of observations = 264, *R*^2^ =.090, *R*^2^ adjusted =.073 when predicting IERQ sum score; number of observations = 263, *R*^2^ =.062, *R*^2^ adjusted =.044 when predicting DIRE excessive reassurance seeking; number of observations = 263, *R*^2^ =.042, *R*^2^ ad justed =.023 when predicting DIRE excessive venting. Significant results *p* < .05 in bold

### Exploratory analyses using abuse and neglect to predict interpersonal ER

To further explore differential effects of childhood abuse versus neglect, three multiple regression models were conducted predicting IERQ sum score, DIRE reassurance-seeking, and DIRE venting using CTQ abuse and neglect scores as predictors. As in the intrapersonal ER analyses, participants with a history of sexual abuse were excluded. Neglect showed significant negative associations with IERQ sum score and DIRE reassurance-seeking, indicating that individuals with greater neglect experiences reported lower use of interpersonal ER strategies. No significant associations were found between childhood abuse and any of the three interpersonal ER outcomes. These results are summarized in Table 3.

In study 2, we aimed to test whether altered intra- and interpersonal ER would predict increased levels of negative mood in daily life, especially in the context of interpersonal stressors.

## Methods study 2

The methods of Study 2 were pre-registered at https://osf.io/tw7mx, and the preregistration protocol was published online Apr 3, 2020. However, the hypotheses tested herein were not pre-registered. All data and code behind this analysis has been made publicly available and can be accessed at https://osf.io/cbkyj/. Study procedures were approved by the ethics committee of the Medical Faculty Mannheim at Heidelberg University (protocol no. 2018–588 N-MA). For study 2, we pooled AA data from a community sample (*n* = 42, reported in 34) and a clinical sample (*n*= 61, reported in 35) that were consecutively recruited within a graduate school on psychosocial consequences of traumatic childhood experiences (www.grk2350.de). The clinical sample was the same sample that we pooled with study 1 for analyzing self-report measures

### Design and material

In both samples, we employed the same AA protocol. The ecological momentary assessment was conducted using the mobile-based platform'movisensXS'app (Movisens GmbH, Karlsruhe, Germany, Version 1.4.8), installed on study smartphones (Moto E, 2nd generation). Participants responded to six pseudo-randomized assessments per day over a period of seven days. At each assessment, they reported on interpersonal problems since the last prompt, as well as momentary mood, which was assessed with the multidimensional mood questionnaire [43]. The MDBF consists of three subscales: energetic arousal, valence, and calmness, each assessed with two bipolar items. Example items include: "Right now I feel: content…discontent, tired…awake". Participants responded on a 1–7 Likert scale, with higher values indicating greater endorsement of the respective mood state. Following [43], we used the valence and calmness subscales to create a composite score for momentary mood, as prior research has demonstrated that these two dimensions are highly interrelated at the between-person level. The MDBF has been validated in previous research and has shown high sensitivity to change, making it a reliable measure for ambulatory assessment [44]. We computed intraclass correlation coefficients (ICCs) to determine the proportion of variance in momentary mood attributable to between-person differences. The ICC(1) value of 0.547 indicates that approximately 54.7% of the variance is due to stable individual differences, whereas the remaining 45.3% reflects within-person fluctuations over time. Furthermore, ICC(2), which reflects the reliability of individual mean scores, was found to be 0.979, suggesting excellent reliability. This indicates that participants’ mean mood values can be interpreted with high confidence as stable trait-level estimates.

Interpersonal problems were assessed at each assessment, using a binary question, "Since the last prompt, did you experience any interpersonal problems or conflicts?" (yes/no). If participants responded "yes", they were prompted to briefly describe the situation in a free-text field and rate its intensity on a scale from 0 (not at all intense) to 100 (extremely intense). For the present analyses, the binary occurrence (yes/no) of interpersonal problems served as the primary variable (see also, [35]). This methodology allows for an ecologically valid measurement of real-life interpersonal stressors, capturing fluctuations in interpersonal problems throughout daily life.

In addition to mood and interpersonal problems, participants in both samples responded to questions on dissociation and trauma-associated intrusions and completed several trials of two behavioral paradigms, all of which are reported elsewhere [34, 35]. Participants received 70 Euros as compensation with an added 30 Euro bonus if they completed more than 90% of prompts. Compliance for the random prompts was high, with participants completing an average of *M* = 38.57 (range 7—42, *SD* = 5.22), prompts per person, resulting in an excellent overall compliance of *M* = 91.84% of random prompts answered (range 16.67%—100%). We did not exclude participants with lower completion rates, as we aimed to maximize data use. Multilevel models effectively handle missing data by estimating parameters based on the available observations, preserving statistical power and minimizing bias.

### Data analysis study 2

To test hypotheses 4 and 5, we conducted a series of multilevel models (MLMs) in R, using the lmer function from the lme4 package [45], and using the lmerTest package [46] to obtain p-values. All MLMs included momentary mood as an outcome, predicted by negative interpersonal events since the last prompt (dummy variable coded as 1 if any event was reported, else 0) and the covariates gender (male = 1, female = 0), time of day centered on noon, weekend (coded as 1 from Friday 5PM to Sunday 5PM, else 0), and study day. The models differed in the person-level predictors included, which were the total score of the DERS in the model testing H4, and the total score of the IERQ in the model testing H5. We modelled their interaction term with negative interpersonal events in predicting mood and included the total score of the CTQ as in indicator for level of CM as an additional covariate. The total scores of the CTQ, DERS and IERQ were centered on the grand mean. All models included random intercepts for each person and random slopes for negative interpersonal events. As suggested by an anonymous reviewer, we included the full mathematical equations for the multilevel models in the supplemental materials (see Supplemental Equations 1 and 2). These equations specify the random effects structure and cross-level interactions tested in H4 and H5, respectively.

## Results study 2

### Sample characteristics study 2

The majority of the *N* = 103 participants in study 2 had a lifetime diagnosis of a mental disorder (75.4%), and previously received a form of outpatient treatment (60.7%.) Detailed clinical diagnoses are presented in Supplemental Table 4. The pooled AA sample comprised 103 individuals who experienced varying levels of CM and psychopathology and completed the same AA protocol. The majority of participants indicated German nationality (97.09%) and were currently living together with their partner or family (40.78%) or in a shared apartment (23.30%). Most were currently single (68.93%) and held a university entrance level degree (85.44%). Supplemental Table 5 in the online supplement provides a detailed overview of demographic variables collected in both samples.

### Self-reported intra-/interpersonal ER and negative affect in daily life

Results for the test of H4 are presented in Supplemental Table 6. In line with H4.1, we observed a significant, negative main effect of the DERS sum score, implying that individuals who reported greater difficulties with intrapersonal ER tended to experience lower momentary mood in daily life. The significant main effect of interpersonal problems indicated that momentary mood was lower at time-points where interpersonal problems were reported than at time-points where such events were not reported. Additionally, and in line with H4.2, we observed a significant interaction effect between the DERS sum score and interpersonal problems as a context factor (see Supplemental Figure 2). This suggests that individuals with higher self-reported intrapersonal ER difficulties experienced lower momentary mood, especially in moments when interpersonal problems were experienced. Both these effects were observed above and beyond any effects of the level of CM, as the CTQ sum score was included in the model as a covariate. The main effect of the CTQ sum score was not significant in this model. Results for the test of H5 are presented in Supplemental Table 7. Contrary to H5.1, interpersonal ER use (IERQ sum score) was not predictive of momentary mood, and neither did it interact with interpersonal problems as a context factor (H5.2). In this model, the level of CM (CTQ sum score) was a significant positive predictor of momentary mood, such that higher levels of CM were associated with more negative momentary mood.

## Discussion

In the studies presented herein, we employed a web-based sample (study 1) for a cross-sectional survey and re-analyzed an existing AA dataset (study 2) to investigate associations between self-reported CM and intrapersonal as well as interpersonal ER. Additionally, we examined whether these ER tendencies predicted negative mood in daily life, particularly in response to interpersonal stressors.

As hypothesized, results showed a positive association between CM severity and difficulties in intrapersonal ER, replicating prior research linking CM to dysregulated emotional patterns [21, 36] regulation strategies [12, 17, 22].

To further probe this association, we tested the differential effects of childhood physical and emotional neglect versus childhood physical and emotional abuse while excluding individuals with a history of sexual abuse, given its well-documented severe impact. Results aligned with prior work, demonstrating that emotional and physical neglect were associated with difficulties in emotional clarity [11] and emotional awareness [10]. Findings are in line with neurobiological assumptions suggesting that the effects of childhood neglect result from a lack of complex, age-appropriate cognitive and social input [15]. In addition to the results for neglect, we replicated previous findings on an association between childhood abuse and impulsivity problems in intrapersonal ER [16, 17]. However, contrary to our hypotheses, we did not find an expected association between neglect and lack of access to effective ER strategies [12–14], but found that abuse predicted these problems.

Given the paucity of evidence on the association between *inter*personal ER and CM experiences, our study is the first to observe that higher levels of CM are associated with reduced use of interpersonal ER (measured with the IERQ). In the present study, we opted to use the IERQ total score instead of its subscales due to their high intercorrelation. Additionally, the German validation study [41] recommends using the total score to minimize redundant variance and enhance interpretability. Greater overall severity of CM was associated with lower levels of the dysfunctional interpersonal emotion regulation strategies ‘excessive reassurance seeking’ and ‘excessive venting’ (DIRE scales). A potential explanation for this could be that individuals who have experienced CM may have repeatedly encountered insensitive, unpredictable, or threatening interactions with their caregivers [21]. As a result, they may be less likely to rely on the assistance and support of others to regulate their emotions – including trying to vent to others about their problems or seeking excessive reassurance. This interpretation remains speculative, as our study did not directly measure trust mechanisms. An alternative perspective is that reduced reliance on interpersonal ER could be adaptive in certain environments where available social support is inconsistent or untrustworthy. Results from additional exploratory analyses appear to support this notion, as they showed that neglect (but not abuse) predicted reduced use of overall interpersonal ER on the IERQ and lower levels of excessive reassurance seeking on the DIRE. These findings suggest that individuals with CM history may generally avoid seeking social support, rather than engaging in dysfunctional interpersonal ER strategies. Future research should directly assess trust mechanisms and examine under what conditions reduced interpersonal ER use may be beneficial versus maladaptive.

In sum, we replicated that a history of CM is associated with problems in intrapersonal ER and conclude that CM (especially neglect) is further associated with lower use of any type of interpersonal ER. Due to the elevated risk of problem behavior among individuals with CM [1, 2] and their increased likelihood of developing mental health problems [17], targeting adaptive intrapersonal and interpersonal ER strategies could serve as a key prevention strategy [47]. Interventions targeted at encouraging functional interpersonal ER attempts, such as describing own feelings to others and asking for the others perspective (co-reappraisal) [48], or asking a close other how they would deal with the situation (co-problem solving) [49], could be valuable additions to existing treatment approaches. In addition to disorder-specific psychotherapy programs such as Dialectical Behavior Therapy [50], an online training program to improve socioemotional skills was positively evaluated [51].

In addition to investigating the association between CM and intra-/interpersonal ER at the between-person level in study 1, in study 2 we tested whether intra-and interpersonal ER tendencies have real life correlates, such that they predict negative mood in the daily lives of individuals with a history of CM. We hypothesized that greater intrapersonal ER difficulties and lower interpersonal ER use would be associated with more negative daily life mood, above and beyond any effects of the level of CM. Importantly, we used interpersonal problems as a context for mood, assuming that ER would be particularly important in moments where interpersonal stressors need to be regulated. Replicating earlier AA work in samples that tend to exhibit high levels of CM, such as individuals with Borderline Personality Disorder [29], we found that momentary mood was more negative at time-points where interpersonal problems were reported. This underlines that interpersonal problems are an important context for negative mood in daily life.

As hypothesized, we also found that individuals with greater self-reported intrapersonal ER difficulties experienced more negative mood in daily life, especially in moments of interpersonal stress. It is important to clarify that Study 2 assessed ER skills as a trait rather than momentary regulatory processes. Thus, our findings indicate that individuals with generally poorer ER skills tend to experience lower daily mood levels, particularly when facing interpersonal stressors. Importantly, this effect remained significant even after controlling for CM severity, suggesting that stable ER tendencies, rather than the level of past CM, play a crucial role in daily mood variability. This highlights intrapersonal ER as a prime intervention target in individuals with CM. For instance, therapies for post-traumatic stress disorder, Borderline Personality Disorder, and depression all target intrapersonal ER, be it through skills use such as in Dialectical Behavior Therapy [50], through cognitive restructuring such as in cognitive behavioral therapy [52], through acceptance based ER strategies in acceptance and commitment therapy [53], or many more.

In contrast to *intra*personal ER, we did not observe significant associations between *inter*personal ER use and daily life mood. In the model testing this association, only the level of CM significantly predicted mood, whereas the IERQ score did not. Thus, in the present sample, we found no direct evidence that the level of mood experienced during moments of interpersonal stress was systematically related to an individual’s general tendency to seek interpersonal support in regulating their emotions. However, there are several possible explanations for this. First, the effect of interpersonal ER may be too weak in light of the predictive power of the level of CM, and this may reflect that interpersonal ER is of lesser importance for daily life mood processes. Second, our measurement approach did not capture the precise timing of interpersonal ER use, as it assessed only self-reported tendencies rather than in-the-moment strategies. It is possible that participants engaged in interpersonal ER before or after reporting mood fluctuations, making it difficult to detect its immediate impact. Future research should consider momentary assessments of interpersonal ER strategies to better understand their temporal relationship with mood fluctuations in daily life.

Third, the measure we used may be inadequate. In contrast to intrapersonal ER *difficulties* measured with the DERS, we did not have an equally suited measure for interpersonal ER difficulties in study 1. We only obtained DIRE scores for participants sampled from a clinical population for the AA sample and hence used the IERQ scores for the analyses, which reflect interpersonal ER *use*, not problems. Beyond this, even the DIRE does not necessarily reflect interpersonal ER difficulties well, or at least not in a sense that may be most relevant to a CM sample. It does not assess, for instance, the difficulties with trusting others that are prevalent in individuals with CM history [54, 55] or problems with actively asking for help, which have been demonstrated in association with barriers to treatment in trauma populations (e.g., [56]). The DIRE rather reflects dysfunctional reaching out to others, but not the issue of reaching out too little or with little skill. Thus, the present results warrant replication with additional measures of interpersonal ER difficulties and there is a clear need for the development of new measurement instruments.

Third, interpersonal ER use may be most relevant in daily life not when interpersonal stressors are present, but when access to interpersonal support is possible. In other words, the interaction partner that was the source of an interpersonal problem is unlikely to also be the person a participant would turn to for interpersonal ER support. Thus, future AA studies should consider assessing the effects of interpersonal ER use at time-points where close others are present or, when they are available in the aftermath of stressful events. In addition to assessing the effects of trait-level interpersonal ER use, assessing momentary interpersonal ER processes would be highly promising. To conclude, the role of interpersonal ER in daily life mood regulation may be highly context-dependent, requiring further investigation into when and how interpersonal ER is beneficial.

Central limitations of both studies pertain to the sampled populations and methods used. First, sample diversity was severely restricted in both samples, which limits the generalizability of the present results to white, cis-female women of a younger age group with a relatively high level of formal education. Future work that assesses intra- and interpersonal ER in cis-men and genderqueer participants is urgently needed, especially considering that trauma type prevalence on the CTQ was shown to differ between cis-women and cis-men [57] and that transgender and gender non-conforming individuals experience higher rates of CM than cis-gender individuals overall, and of emotional neglect in specific [58]. Additionally, a broader age range of participants is needed in future studies, to test whether the effects of CM that lie further in the past (i.e. in older individuals) wear off in time. In any case, CM timing and duration should be assessed in future work as they may explain how severely ER development is affected.

Second, a key limitation of Study 2 is that emotion regulation skills were assessed as a stable trait rather than as momentary regulatory processes. While this approach allows for an investigation of general ER tendencies, it does not capture the dynamic regulation attempts individuals make in response to daily stressors. Future research should incorporate ambulatory assessments that allow for real-time tracking of interpersonal ER strategies. This would help clarify whether interpersonal ER occurs as an immediate response to stress, whether its effects on mood unfold over time, or whether individuals engage in interpersonal ER before or after reporting mood changes. Additionally, momentary assessments could differentiate between proactive and reactive interpersonal ER—i.e., whether individuals seek social support in anticipation of distress or only after experiencing heightened negative affect. Investigating these temporal aspects of interpersonal ER in daily life could provide a more nuanced understanding of its regulatory function and inform targeted mental health interventions.

Third, as discussed briefly above, the assessment of interpersonal ER somewhat limits the conclusions that can be drawn from the present results. First, the IERQ [40] only assesses the level of interpersonal ER use, but not difficulties with interpersonal ER. In other words, it assesses a preference for turning to others for ER support but not how easily this behavior comes to an individual or how successful it is. As previous evidence shows that individuals who experienced CM tend to perceive others as threatening and have problems with trusting others [55, 59, 60], it seems likely that both selecting a (trusted) person to seek out for interpersonal ER and then processing the offered response could be problematic. In addition to the IERQ, we included the DIRE questionnaire, which does assess problems with interpersonal ER, but only in the specific sense of using the strategies venting and reassurance seeking too excessively. Again, using interpersonal ER too much may not be the central problem for individuals with CM, but rather choosing which person to trust for receiving ER help, overcoming the fear of asking for help, and then processing what is offered are likely to be focal difficulties. Thus, much of the intricacies of interpersonal ER remain unclear and we hope that future work can build on the results presented herein to further disentangle this process. In order to do this, additional self-report measures for cross-sectional studies are needed, as is further AA work to assess the process of interpersonal ER in daily life at a fine-grained timescale.

Lastly, in both studies, causal relationships between CM and intra- and interpersonal ER cannot be assumed and the observed results are at present only of an observational, correlational nature. To test whether and how exactly CM impairs interpersonal ER development, longitudinal studies are needed that follow children who experienced CM throughout a longer developmental period. In addition to this, quasi-experimental studies where individuals with various levels of CM can choose interpersonal ER to regulate experimentally induced negative affect would be helpful for understanding the CM-interpersonal ER association.

## Conclusions

The present studies replicated the well-documented association between CM and intrapersonal ER difficulties and augmented this by demonstrating associations between neglect and a lack of emotional awareness or clarity versus an association between abuse and impulsivity during intrapersonal ER. Additionally, we demonstrated that individuals with greater self-reported intrapersonal ER difficulties tended to experience lower daily mood levels, particularly in moments of interpersonal stress. However, it is important to note that ER strategies were assessed as stable traits rather than dynamically tracked in daily life. Beyond investigating self-reported *intra*personal ER, this study was the first to test associations between CM and *inter*personal ER. Results indicated reduced self-reported use of interpersonal ER in general, including reduced levels of excessive venting or reassurance seeking, suggesting that individuals who experienced CM may be less inclined to seek support from others in general (rather than seeking support in a dysfunctional way). However, this reduction in interpersonal ER use may not necessarily be maladaptive. Future research should investigate the specific contexts in which interpersonal ER avoidance may serve as an adaptive coping strategy. This behavioral pattern may provide additional insight into the development of interpersonal problems in later life, and facilitate the development of preventative and therapeutic intervention strategies.

## Supplementary Information

Supplementary Material 1.

## Data Availability

All data and code behind this analysis has been made publicly available and can be accessed at https://osf.io/cbkyj/.

## Footnotes

1 As suggested by an anonymous reviewer, we repeated this analysis without participants who reported sexual abuse, analyzing the differential associations of neglect versus abuse with the CTQ sum score in *n* = 294 participants. Results indicated that both trauma types, neglect and abuse, remained significantly associated with the CTQ sum score.
