# Supplementary material for: Intra- and interpersonal emotion regulation are altered in individuals with childhood maltreatment: cross-sectional associations and effects on daily life mood
Source: Borderline Personal Disord Emot Dysregul. 2025 Jun 17;12:23. doi: 10.1186/s40479-025-00297-0 (PMC12172324; doi:10.1186/s40479-025-00297-0)
Supplement: Supplementary file 1 — Supplementary Material 1. [file 40479_2025_297_MOESM1_ESM.pdf]

*Supplemental Table 1.* Descriptive results for study 1.

| | *M* | *SD* | *Md* | *Min* | *Max* | *Skew* |
|---|---|---|---|---|---|---|
| **Childhood maltreatment** | | | | | | |
| CTQ total | 53.46 | 19.30 | 51 | 25 | 120 | 0.84 |
| CTQ abuse | 21.52 | 8.85 | 20 | 10 | 49 | 0.87 |
| CTQ neglect | 23.69 | 8.64 | 23 | 10 | 50 | 0.37 |
| CTQ sexual abuse | 8.25 | 5.32 | 5 | 5 | 25 | 1.88 |
| CTQ physical abuse | 7.57 | 4.08 | 6 | 5 | 24 | 1.95 |
| CTQ emotional abuse | 13.95 | 5.83 | 14 | 5 | 25 | 0.22 |
| CTQ physical neglect | 9.10 | 3.72 | 8 | 5 | 25 | 1.14 |
| CTQ emotional neglect | 14.59 | 5.69 | 15 | 5 | 25 | 0.03 |
| | | | | | | |
| **Intrapersonal ER difficulties** | | | | | | |
| DERS total | 102.71 | 28.10 | 103 | 46 | 171 | -0.02 |
| DERS impulsivity in ER | 14.16 | 5.96 | 13 | 6 | 30 | 0.62 |
| DERS lack of strategies | 22.74 | 7.98 | 23 | 8 | 40 | 0.09 |
| DERS lack of awareness | 17.16 | 5.49 | 17 | 6 | 30 | 0.18 |
| DERS lack of clarity | 13.03 | 4.60 | 12 | 5 | 25 | 0.35 |
| DERS nonacceptance | 18.01 | 6.77 | 18 | 6 | 30 | 0.07 |
| DERS difficulty in goals | 17.63 | 4.90 | 18 | 5 | 25 | -0.31 |
| | | | | | | |
| **Interpersonal ER difficulties** | | | | | | |
| DIRE acceptance | 8.40 | 3.37 | 8 | 3 | 15 | 0.09 |
| DIRE avoidance | 18.71 | 5.54 | 18 | 6 | 30 | 0.03 |
| DIRE reassurance seeking | 13.22 | 5.62 | 13 | 6 | 30 | 0.55 |
| DIRE venting | 14.85 | 4.91 | 14 | 6 | 29 | 0.31 |
| | | | | | | |
| **Interpersonal ER use** | | | | | | |
| IERQ total | 58.89 | 13.08 | 60 | 20 | 99 | -0.17 |
| IERQ positive affect | 18.20 | 4.47 | 19 | 5 | 25 | -0.61 |
| IERQ perspective taking | 11.44 | 3.60 | 11 | 5 | 25 | 0.55 |
| IERQ soothing | 13.30 | 4.56 | 13 | 5 | 25 | 0.15 |
| IERQ social modeling | 15.94 | 4.62 | 16 | 5 | 25 | -0.29 |

*Supplemental Table 2.* Linear multiple regression model for the test of H1.

| | DERS sum score | | | | |
|---|---|---|---|---|---|
| *Predictors* | *Est.* | *beta* | *SE* | *CI* | *p* |
| Intercept | 75.67 | 0.00 | 6.23 | 63.44;87.91 | **<.001** |
| CTQ sum | 0.66 | 0.44 | 0.06 | 0.54;0.78 | **<.001** |
| Education | -0.14 | -0.00 | 2.96 | -5.96;5.68 | .962 |
| Gender | -13.22 | -0.11 | 4.64 | -22.34;-4.10 | **.005** |
| Age | -0.21 | -0.07 | 0.12 | -0.46;0.03 | .084 |

*Note.* Number of observations = 491. $R^2$ = .221, $R^2$ adjusted = .214.

*Supplemental Table 3*. Linear multiple regression models for the test of H3.

| | IERQ sum score | | | | DIRE excessive reassurance seeking | | | | DIRE excessive venting | | | |
|---|---|---|---|---|---|---|---|---|---|---|---|---|
| *Predictors* | *Est.* | *beta* | *SE* | *p* | *Est.* | *beta* | *SE* | *p* | *Est.* | *beta* | *SE* | *p* |
| Intercept | **71.95** | **0.00** | **2.96** | **<0.001** | **17.86** | **0.00** | **1.38** | **<0.001** | **16.62** | **0.00** | **1.20** | **<0.001** |
| CTQ sum score | **-0.25** | **-0.37** | **0.03** | **<0.001** | **-0.06** | **-0.19** | **0.01** | **<0.001** | **-0.06** | **-0.23** | **0.01** | **<0.001** |
| Education | 0.13 | 0.00 | 1.41 | 0.926 | 0.62 | 0.04 | 0.66 | 0.347 | 0.03 | 0.00 | 0.57 | 0.954 |
| Gender | -3.76 | -0.07 | 2.21 | 0.090 | -0.05 | -0.00 | 1.02 | 0.958 | -0.07 | -0.00 | 0.89 | 0.940 |
| Age | -0.00 | -0.00 | 0.06 | 0.972 | **-0.07** | **-0.12** | **0.03** | **0.010** | 0.05 | 0.09 | 0.02 | 0.050 |

*Note.* Number of observations = 491, $R^2$ = .142, $R^2$ adjusted = .135 when predicting IERQ sum score; number of observations = 489, $R^2$ = .056, $R^2$ adjusted = .049 when predicting DIRE excessive reassurance seeking; number of observations = 490, $R^2$ = .062, $R^2$ adjusted = .055 when predicting DIRE excessive venting.

*Supplemental Table 4*. Demographic data for participants included in study 2 (n = 103).

| | N | % |
|---|---|---|
| **Nationality** | | |
| German | 100 | 97.09 |
| German-Italian | 1 | 0.97 |
| German-Chilean | 1 | 0.97 |
| Austrian | 1 | 0.97 |
| **Housing situation** | | |
| Alone | 31 | 30.10 |
| With partner/ own family | 42 | 40.78 |
| With parents | 5 | 4.85 |
| Shared apartment | 24 | 23.30 |
| No regular living conditions | 1 | 0.97 |
| **Relationship status** | | |
| Single | 71 | 68.93 |
| Married | 16 | 15.53 |
| Long-term relationship | 8 | 7.77 |
| Divorced | 7 | 6.80 |
| Widowed | 1 | 0.97 |
| **Formal education** | | |
| Graduation after 9 yrs | 2 | 1.94 |
| Graduation after 10 yrs | 13 | 12.62 |
| Graduation after 12-13 yrs | 88 | 85.44 |

*Note.* Graduation after 9 years = German “Hauptschulabschluss”, Graduation after 10 years = German “Realschulabschluss”, graduation after 12-13 years = German “Abitur” (university entrance level degree).

*Supplemental Table 5.* DSM-5 disorders of the clinical participants (N=61) included in sample 2 according to SCID-5-CV.

| | **Current** | | **Lifetime** | |
|---|---|---|---|---|
| | **N** | **%** | **N** | **%** |
| No lifetime diagnosis according to SCID-5-CV | – | – | 15 | 24.6 |
| Posttraumatic Stress Disorder | 17 | 27.9 | 21 | 34.4 |
| Major Depressive Disorder, Single Episode | 1 | 1.6 | 8 | 13.1 |
| Major Depressive Disorder, Recurrent Episode | 11 | 18.0 | 28 | 44.3 |
| Persistent Depressive Disorder | 5 | 8.2 | 5 | 8.2 |
| Brief Psychotic Disorder | 0 | 0.0 | 1 | 1.6 |
| Substance Use Disorders (SUD) | 3 | 4.9 | 16 | 26.2 |
| Panic Disorder/ Agoraphobia | 1 | 1.6 | 5 | 8.2 |
| Social Anxiety Disorder | 8 | 13.1 | 12 | 18.0 |
| Generalized Anxiety Disorder | 1 | 1.6 | 1 | 1.6 |
| Specific Phobia | 3 | 4.9 | 3 | 4.9 |
| Obsessive-Compulsive Disorder | 1 | 1.6 | 2 | 3.3 |
| Anorexia Nervosa | 0 | 0.0 | 6 | 9.8 |
| Bulimia Nervosa | 1 | 1.6 | 6 | 9.8 |
| Somatic Symptom Disorder | 6 | 9.8 | 6 | 9.8 |
| Other DSM-5 Disorder | 2 | 1.6 | 5 | 8.2 |

*Note.* In case of a mild SUD, abstinence of at least two months was required; a moderate/ severe SUD required abstinence of at least 12 months.

*Supplemental Equation 1.* Mathematical equation for the multilevel model testing H4. The model includes random intercepts and slopes for negative interpersonal event incidence and specifies the cross-level interaction with DERS scores.

**Level-1 Model (within-person variation):**

$$Affect_{ti} = \beta_{0i} + \beta_{1i} \cdot ip.neg.dic_{ti} + \beta_2 \cdot gender_i + \beta_3 \cdot weekend_{ti} + \beta_4 \cdot time.of.day_{ti} + \beta_5 \cdot studyday_{ti} + \varepsilon_{ti}$$

**Level-2 Model (between-person differences):**

$$\beta_{0i} = \gamma_{00} + \gamma_{01} \cdot DERS_i + \gamma_{02} \cdot CTQ_i + u_{0i}$$

$$\beta_{1i} = \gamma_{10} + \gamma_{11} \cdot DERS_i + u_{1i}$$

**Full Model Equation:**

$$Affect_{ti} = \gamma_{00} + \gamma_{01} \cdot DERS_i + \gamma_{02} \cdot CTQ_i + \gamma_{10} \cdot ip.neg.dic_{ti} + \gamma_{11} \cdot ip.neg.dic_{ti} \cdot DERS_i + \beta_2 \cdot gender_i + \beta_3 \cdot weekend_{ti} + \beta_4 \cdot time.of.day_{ti} + \beta_5 \cdot studyday_{ti} + u_{0i} + u_{1i} \cdot ip.neg.dic_{ti} + \varepsilon_{ti}$$

*Supplemental Equation 2.* Mathematical equation for the multilevel model testing H5. The model includes random intercepts and slopes for negative interpersonal event incidence and specifies the cross-level interaction with IERQ scores.

**Level-1 Model (within-person variation):**

$$Affect_{ti} = \beta_{0i} + \beta_{1i} \cdot ip.neg.dic_{ti} + \beta_2 \cdot gender_i + \beta_3 \cdot weekend_{ti} + \beta_4 \cdot time.of.day_{ti} + \beta_5 \cdot studyday_{ti} + \varepsilon_{ti}$$

**Level-2 Model (between-person differences):**

$$\beta_{0i} = \gamma_{00} + \gamma_{01} \cdot IERQ_i + \gamma_{02} \cdot CTQ_i + u_{0i}$$

$$\beta_{1i} = \gamma_{10} + \gamma_{11} \cdot IERQ_i + u_{1i}$$

**Full Model Equation:**

$$Affect_{ti} = \gamma_{00} + \gamma_{01} \cdot IERQ_i + \gamma_{02} \cdot CTQ_i + \gamma_{10} \cdot ip.neg.dic_{ti} + \gamma_{11} \cdot ip.neg.dic_{ti} \cdot IERQ_i + \beta_2 \cdot gender_i + \beta_3 \cdot weekend_{ti} + \beta_4 \cdot time.of.day_{ti} + \beta_5 \cdot studyday_{ti} + u_{0i} + u_{1i} \cdot ip.neg.dic_{ti} + \varepsilon_{ti}$$

*Supplemental Table 6.* Multilevel model testing H4; momentary mood predicted with negative interpersonal events, intrapersonal ER, CM, and covariates.

| | **Momentary Mood** | | | | |
|---|---|---|---|---|---|
| *Predictors* | *Est.* | *beta* | *SE* | *CI* | *p* |
| Intercept | 3.72 | -0.00 | 0.09 | 3.55;3.90 | **<0.001** |
| Neg. interpers. Event | -0.52 | -0.13 | 0.06 | -0.65;-0.39 | **<0.001** |
| DERS sum | -0.02 | -0.47 | 0.00 | -0.03;-0.02 | **<0.001** |
| CTQ sum | -0.00 | -0.03 | 0.00 | -0.01;0.01 | 0.715 |
| Gender | -0.35 | -0.06 | 0.30 | -0.93;0.24 | 0.243 |
| Weekend | 0.21 | 0.07 | 0.03 | 0.15;0.27 | **<0.001** |
| Time of Day | 0.01 | 0.04 | 0.00 | 0.01;0.02 | **<0.001** |
| Study Day | 0.01 | 0.02 | 0.01 | -0.00;0.03 | 0.067 |
| Neg. interpers. event × DERS | -0.00 | -0.04 | 0.00 | -0.01;-0.00 | **0.028** |

*Note*. N observations = 3963, n individuals = 103. ICC = 0.43.

*Supplemental Table 7.* Multilevel model testing H5; momentary mood predicted with negative interpersonal events, IERQ, and covariates.

| | **Momentary Mood** | | | | |
|---|---|---|---|---|---|
| *Predictors* | *Est.* | *beta* | *SE* | *CI* | *p* |
| Intercept | 3.70 | -0.01 | 0.10 | 3.50;3.90 | **<0.001** |
| Neg. interpersonal event | -0.55 | -0.14 | 0.07 | -0.68;-0.42 | **<0.001** |
| IERQ sum | 0.01 | 0.10 | 0.01 | -0.00;0.02 | 0.138 |
| CTQ sum | -0.02 | -0.26 | 0.00 | -0.03;-0.01 | **<0.001** |
| Gender | -0.04 | -0.01 | 0.38 | -0.79;0.70 | 0.910 |
| Weekend | 0.21 | 0.07 | 0.03 | 0.15;0.27 | **<0.001** |
| Time of Day | 0.01 | 0.04 | 0.00 | 0.01;0.02 | **<0.001** |
| Study Day | 0.01 | 0.02 | 0.01 | -0.00;0.03 | 0.068 |
| Neg. interpersonal event × IERQ sum | -0.01 | -0.02 | 0.00 | -0.01;0.00 | 0.267 |

*Note*. N observations = 3963, n individuals = 103. ICC = 0.52.

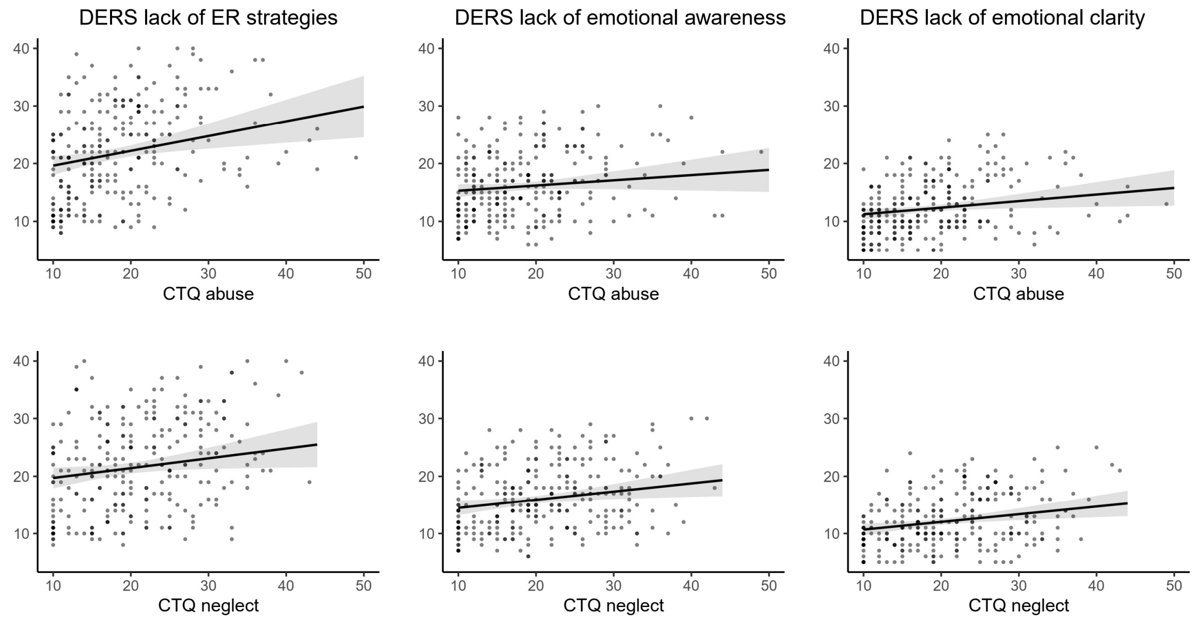


*Supplemental Figure 1.* Illustration of results for H2, association between subscales of the Childhood Trauma Questionnaire (CTQ) and subscales of the Difficulties in Emotion Regulation Scale (DERS).

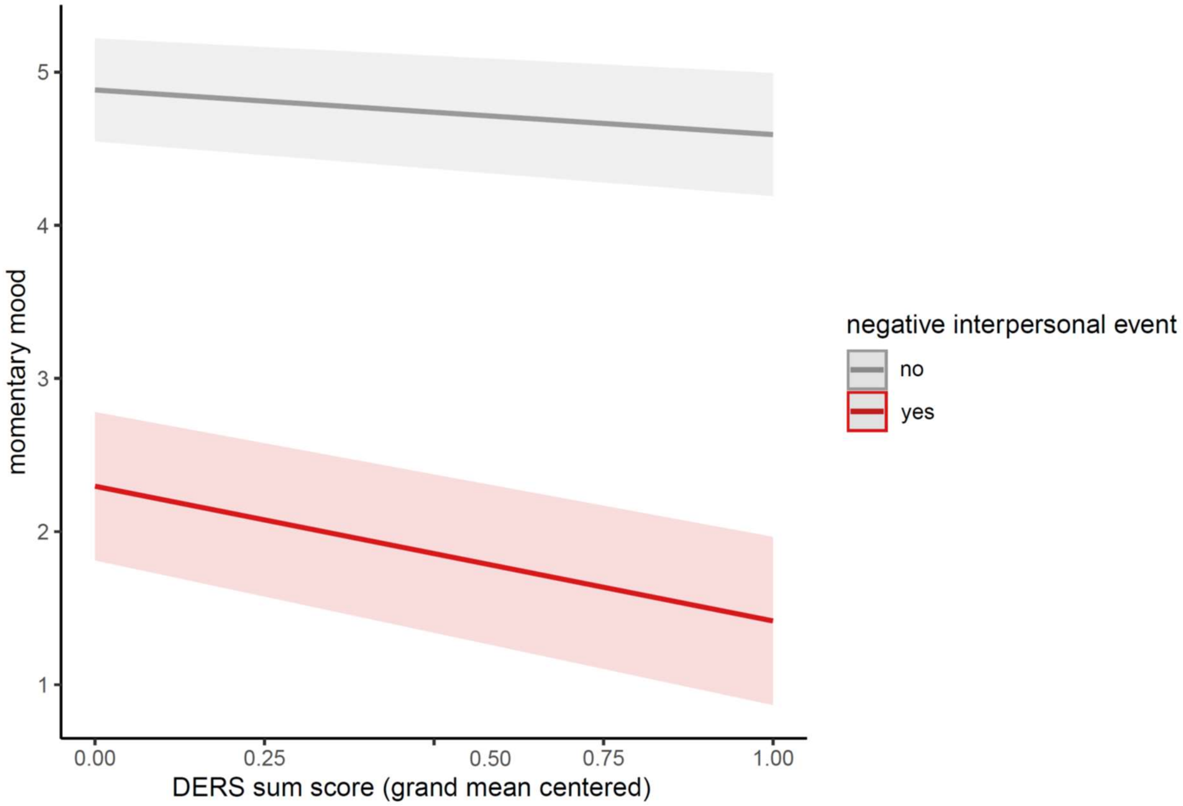


*Supplemental Figure 2.* Illustration of the interaction effect between Difficulties in Emotion Regulation Scale (DERS) sum score and interpersonal problems (Hypothesis 4.2).

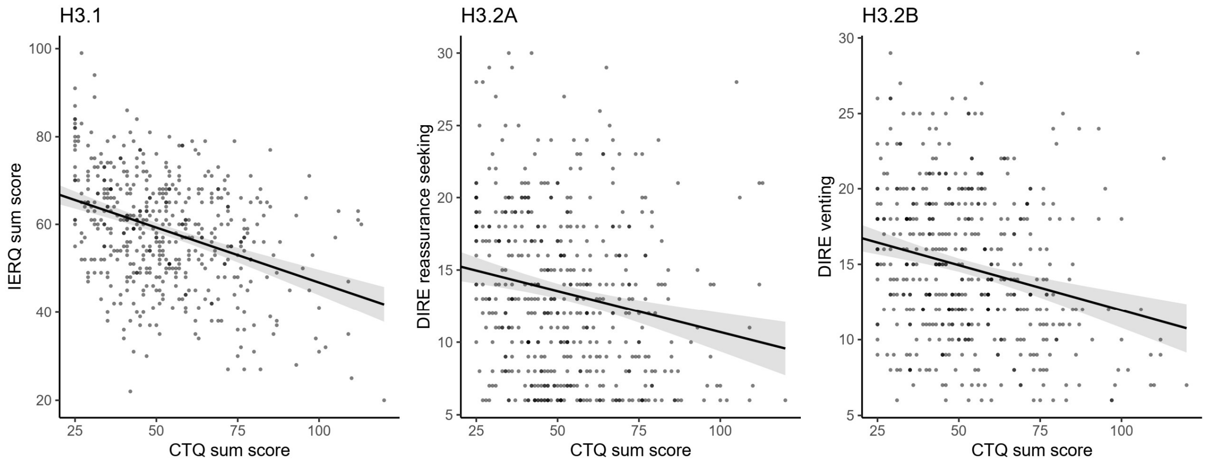


*Supplemental Figure 2.* Illustration of results for H3, association between sum score of the Childhood Trauma Questionnaire (CTQ), the Interpersonal Emotion Regulation Questionnaire (IERQ), and subscales of the Difficulties in Interpersonal Regulation of Emotions (DIRE).
